# The Effect of p38 Mitogen-Activated Protein Kinase Activation on Inflammatory Liver Damage following Hemorrhagic Shock in Rats

**DOI:** 10.1371/journal.pone.0030124

**Published:** 2012-01-09

**Authors:** Hiroaki Sato, Toshiko Tanaka, Noriyuki Tanaka

**Affiliations:** Department of Forensic Medicine, University of Occupational and Environmental Health, Japan, Yahata-Nishi, Kitakyushu, Japan; University of Maryland School of Pharmacy, United States of America

## Abstract

Hemorrhagic shock is a frequent cause of liver failure and often leads to a fatal outcome. Several studies have revealed that p38 MAPK is a key mediator in hemorrhagic damage of the primary organs through the activation of proinflammatory cytokines such as tumor necrosis factor (TNF)-α and interleukin (IL)-1β. However, the precise role of these factors in liver damage following hemorrhagic shock is unclear. In this study, we used FR167653, a specific inhibitor of p38 MAPK phosphorylation, to examine the role of p38 MAPK in liver damage occurring up to 5 hours after a hemorrhagic episode in a rat model. Activation of p38 MAPK in the liver as well as an increase in hepatic mRNA expression and serum concentrations of TNF-α and IL-1β occurred during the early phase after hemorrhage. Increased serum levels of hepatic enzymes, as well as histological damage and activated neutrophil accumulation in the liver, were observed in the late phase following hemorrhagic shock. FR167653 inhibited the inflammation-related hepatic injury following hemorrhagic shock. Bacterial lipopolysaccharide (LPS) derived from the gut appeared to have little effects on the hepatic damage. These results demonstrate that p38 MAPK activation is induced by hepatic ischemia during hemorrhagic shock and plays an important role both in the hepatic expression of proinflammatory cytokines and in the development of inflammation-related liver damage.

## Introduction

The liver, which plays a crucial role in metabolism and homeostasis, is one of the organs that is most susceptible to injury arising from hemorrhagic shock, the outcome of which is often fatal [1]. Several studies on ischemic-reperfusion injury of the liver have suggested that p38 MAPK plays a pivotal role in the progression of hemorrhage-induced damage [2], [3], [4], [5], [6]. p38 MAPK has been postulated to be one of the key factors promoting the expression of pro-inflammatory cytokines such as TNF-α and IL-1β [7]. These cytokines play an important role in inflammatory organ damage by promoting the recruitment of neutrophils which release reactive oxygen species and proteases [8], [9], [10]. Ischemia and ischemia-reperfusion of organs are consequences of systemic hemodynamic changes following hemorrhagic shock [10], [11], [12], [13]. We previously hypothesized that p38 MAPK activation might play an important role in the progression of liver damage following hemorrhagic shock, but this possibility was not tested experimentally.

In this study, the role of p38 MAPK activation in the development of inflammatory liver damage following hemorrhagic shock was examined by the use of FR167653, which is a specific inhibitor of p38 MAPK phosphorylation. Our experimental model provided a novel paradigm to explore the cause of liver damage following hemorrhagic shock.

## Materials and Methods

### Animals

Male Wistar rats (Seiwa Experimental Animal Co.; Oita, Japan) weighing 260 g to 350 g were used. The rats were allowed to acclimate and were maintained at 22°C with 12 hours light-dark cycles for 2 weeks with ad libitum access to water and standard rat feed. The animals were fasted overnight (with ad libitum access to water) prior to the experiments so as to minimize the possible influence of feeding on factors such as the vomiting reflex, hemodynamic responses, and other unexpected reactions. The Ethics Committee of Animal Care and Experimentation, University of Occupational and Environmental Health, Japan, approved all requests for animals and the intended procedures of the present study (Permission numbers: AE 07-029) according to the Guide for the Care and Use of Laboratory Animals published by the US National Institute of Health (NIH Publication No 85-23, revised 1996).

### Hemorrhage procedure

Animals were anesthetized using a mixture of urethane (ethyl carbamate; 470 mg/kg body weight) and α-chloralose (23 mg/kg body weight) administered by intraperitoneal injection with touchup dosing as needed to maintain a sufficient depth of anesthesia throughout the experimental period. The animals were placed in the supine position on a temperature-controlled surgical board (37±1°C) and allowed to breathe spontaneously. After the induction of anesthesia, the left femoral artery was cannulated under aseptic conditions with a 3 Fr polyethylene catheter (Atom Medical Co., Tokyo, Japan) connected to a blood pressure transducer (G-1000, Nihon Kohden, Tokyo, Japan) and a polygraph (LEG-1000, Nihon Kohden, Tokyo, Japan) for blood pressure monitoring. Heparinized saline (700 U/ml) was used to fill the cannula prior to placement and to prevent systemic coagulation after placement. The hepatic artery was exposed via a midline laparotomy and a flow probe with LBF–III laser Doppler flow meter (Biomedical Science, Kanazawa, Japan) was placed on the artery. Hepatic arterial blood flow (HBF) and mean arterial blood pressure (MBP) were measured every 20 minutes. The HBF values of the controls (Sham group) were expressed as a ratio relative to the average values at time 0, and those of the hemorrhage groups were expressed as a ratio relative to the average values of the Sham group at each timepoint. After an equilibrium period, the rats were bled via the catheter using a syringe at a constant rate for 20 minutes. Up to 25% (i.e. 1.625 ml/100 g) of the total body blood volume (6.5±0.1ml/100 g body weight) [14] was drawn.

Prior to conducting the main experiment, we investigated the effect of bleeding volume on the 5-hour-mortality rate. Following bleeding of 33% of the total blood volume, the mortality rate was 50%; failure of the cardiovascular system occurred promptly and was followed immediately by death or a near-death state while under anesthesia. With bleeding of up to 25% of the total blood volume, few animals died but most suffered from hemorrhagic shock during the first 5 hours under anesthesia. To maximize experimental reproducibility and stability, 25% bleeding was employed in all subsequent experiments.

Many studies have investigated hemorrhagic shock using artificial continuous long-term hypotension and fluid resuscitation models [15], [16]. Tsukamoto et al. reported that fixed-pressure hemorrhage models were reproducible but did not correspond with clinical cases in which the shock episode was not sustained for a sufficiently long duration as a result of various autoregulatory reactions, such as tachycardia, vasoconstriction, oliguria, and absorption of interstitial fluid into the blood [17]. Therefore, in this study, we used a bleeding model rather that the artificial sustained hypotension and fluid re-infusion model for investigating the intrinsic pathophysiological processes following hemorrhagic shock.

### Experimental protocol

Rats were divided into 4 treatment groups. Non-bled rats treated with physiological saline served as negative controls (Sham group), while rats that underwent hemorrhage and were treated with saline served as positive controls (Hemorrhage group). In addition, to determine whether p38 MAPK activation affects the inflammatory hepatic injury following hemorrhagic shock, rats treated with FR167653 (FR) were subjected to sham (FR group) or hemorrhage (FR+Hemorrhage group) procedures. After recording MBP and HBF, the rats in each group were sacrificed by exsanguination from punctured hepatic and portal veins, and the livers were collected at 1, 3 or 5 hours after bleeding (n = 5 in each group except in the MAPK assay). Serum was separated by centrifuging the blood samples, collected from the hepatic and portal veins, at 3,000 rpm for 20 minutes. The tissue and serum samples were stored at –80°C until assayed.

FR, {1-[7-(4-fluorophenyl)-1,2,3,4-tetrahydro-8-(4-pyridyl)pyrazolo[5,1-c](1,2,4) triazin–2-yl]-2-phenylethanedine sulfate monohydrate}(Astellas Pharma Inc., Tokyo, Japan), is a novel synthetic compound that prevents p38 MAPK activation by suppressing p38 MAPK phosphorylation [4], [18]. FR has frequently been used to study the role of p38 MAPK activation [4], [5], [19], [20]. FR was dissolved in physiological saline at a concentration of 5 mg/ml and administered at a dose of 5 mg/kg body weight. Several studies have reported the use of FR167653 at doses of 1 to 10 mg/kg body weight without causing liver damage as a side effect [4], [5], [20]. FR or physiological saline was administered by intramuscular injection in the anterior region of the right thigh 30 minutes after the cannulation, and bleeding was started 30 minutes after intramuscular injection.

### Bacterial LPS concentration in the portal vein

Whole blood samples collected from the portal vein were centrifuged at 3,000 rpm for 10 minutes, and the separated plasma was used for the assay. The high-sensitivity analysis was carried out by kinetic turbidimetric Limulus assay using a MT-251 Toxinometer (Wako, Osaka, Japan), which theoretically can measure LPS at concentrations as low as 0.01pg/mL.

### Assessment of p38 MAPK activation in the liver

Western blot analysis was performed as described previously [12], [13]. Briefly, liver tissue was homogenized with extraction buffer (10 mM Tris, pH 8.0, 5 mM EDTA, 50 mM NaCl, 30 mM sodium pyrophosphate, 50 mM sodium fluoride, and 100 µM sodium vanadate) containing protease inhibitors cocktail. After centrifugation at 18,000xg for 20 minutes, the protein concentration in the upper layer was measured; 20 µg of the lysate was then resolved by SDS-PAGE and transferred to PVDF membranes (Immobilon, Millipore Corp, Bedford, MA, USA). The membrane was blocked with TBS buffer (10 mM Tris-Cl, pH 7.4, 0.15 M NaCl) containing 5% nonfat dried milk, then reacted with rabbit polyclonal phospho-p38 MAPK antibody or rabbit polyclonal p38 MAPK antibody (1∶1000 dilution; New England Biolabs, Beverly, MA, USA) at 4°C for 16 hours. After incubation with goat anti-rabbit IgG peroxidase conjugate (1∶1000 dilution; DAKO, Glostrup, Denmark) for 1 hour at 25°C, the immune complex was visualized using enhanced chemiluminescence (Amersham Bioscience, Piscataway, NJ, USA). The densities of the bands were quantified using Scion Image Beta 4.02 (Scion Corporation, Frederick, MA, USA) and the ratio of phospholyl/non-phospholyl p38 MAPK was calculated. The values were expressed as a ratio relative to the average value for the Sham group at each timepoint.

### Assessment of TNF-α and IL-1β mRNA expression in the liver

Total RNA was extracted from each liver using ISOGEN (Nippon gene, Toyama, Japan). A 1 µg aliquot of total RNA from liver was reverse-transcribed using RNase H-reverse Transcription and random hexamers (Invitrogen, CA, USA). To assess the amount of TNF-α and IL-1β mRNA in each sample, polymerized chain reaction (PCR) was performed for TNF-α, IL-1βand glyceraldehyde-3-phosphate dehydrogenase (GAPDH), the latter being a constitutively expressed housekeeping gene. PCR was performed in 5.0 µL of PCR sample using the AmplitTaq Gold 360 Master Mix (Applied Biosytems, CA, USA). PCR products were separated by electrophoresis using 1% agarose gel containing 0.3 µg/ml ethidium bromide to visualize DNA bands, and the gels were scanned using a Mupid-Scope WD (Advance, Tokyo, Japan). Densitometry was performed with Adobe Photoshop software (Adobe Systems Inc, Mountain View, CA, USA). The densities of the bands were quantified using Scion Image Beta 4.02 (Scion Corporation, MA, USA) and the ratio of TNF-α or IL-1β/GAPDH mRNA was calculated. The values were expressed as a ratio as described above for p38 MAPK activation.

### Hepatic serum TNF-α and IL-1β concentration

The concentrations of TNF-α and IL-1β in serum collected from the hepatic vein were measured using rat TNF-α and IL-1β enzyme linked immunosorbant assay kits (Invitrogen, CA, USA).

### Liver enzymes analysis

The serum levels of aspartate aminotransferase (AST) and alanine aminotransferase (ALT) were measured to assess damage to the hepatic parenchyma. Blood samples were processed in a Hitachi 7350 automatic analyzer (Hitachi Ltd., Tokyo, Japan) and enzyme levels were expressed as units per liter.

### Histological examination

For light microscopic examination, the right lobe of the liver was cut into 2-mm-thick slices and fixed with 2.5% glutaraldehyde in 0.1 M phosphate-buffered saline (PBS), pH 7.6. Dewaxed sections were incubated in 3% hydrogen peroxide for 10 min and washed in 0.1 M PBS, pH 7.6. Rabbit polyclonal anti-rat myeloperoxidase (MPO) antibody as a marker for neutrophils (Laboratory Vision Co., CA, USA) was applied to the sections, which were then incubated at room temperature for 1 hour. Products resulting from the immunoreaction were visualized by the peroxidase-conjugated streptavidin-biotin method (Simple-Stain-PO kit; Nichirei Corp. Tokyo, Japan) using 3,3′-diaminobenzidine tetrahydrochloride (DAB) and hydrogen peroxide. The nuclei were counterstained with hematoxylin. As a control for immunostaining, Tris buffer or normal sera were substituted for the primary antibodies.

Histological changes were assessed and MPO-positive cells were counted in 10 randomly selected fields per liver (500 µm^2^) for 5 rats in each group. The MPO data were expressed as the number of positive cells per field. All histological analyses were performed by a pathologist without prior knowledge of the experimental conditions.

### Statistical analysis

Data are expressed as mean±SE. Differences among groups were tested for statistical significance using one-way analysis of variance and Fisher post hoc testing. Data correlations were tested using Pearson's correlation coefficient test. A p value of less than 0.05 was regarded as a statistically significant difference.

## Results

### Changes in systemic blood pressure and hepatic blood flow caused by hemorrhage

**Figure 1:**
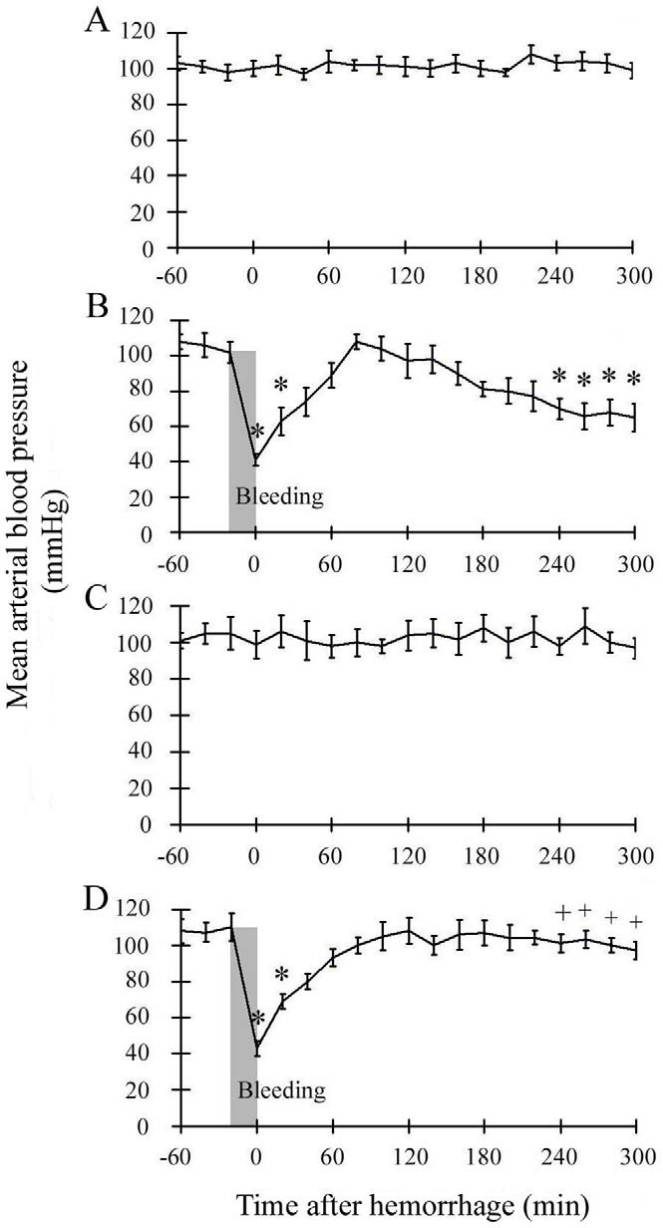
Time course of mean arterial blood pressure (MBP). Hemorrhage was induced for 20 minutes (shaded area). A: Sham group (Sham), B: Hemorrhage group (Hemorrhage), C: Sham with FR167653 treatment (FR), D: Hemorrhage with FR167653 treatment (FR+Hemorrhage). MBP decreased significantly just after hemorrhage and returned to baseline at 40 minutes, but decreased gradually in the latter phase in the Hemorrhage group (B). FR treatment did not have any effect on the primary MBP decrease, although it abolished the secondary MBP decrease after hemorrhage (D). Data are shown as mean±SE. n = 5/groups, * p<0.05 vs Sham, + p<0.05 between Hemorrhage and FR+Hemorrhage.

There were no significant differences in MBP between the Sham group and FR group throughout the experiment (Fig. 1A, Fig. 1C). MBP significantly decreased immediately after a hemorrhage as shown in Fig. 1B and Fig. 1D, (from 102 ±6 mmHg to 41±3 mmHg in the Hemorrhage group; from 110±8 mmHg to 43±4 mmHg in the FR+Hemorrhage group) and returned to the baseline level at 40 minutes after hemorrhage in both groups. In the latter phase after hemorrhage, MBP tended to decrease gradually over time and was significantly lower in the Hemorrhage group than in the Sham group at 240 minutes (Fig. 1B), although MBP in the FR+Hemorrhage group did not show a decrease in MBP during the latter phase after hemorrhage (Fig. 1D).

**Figure 2:**
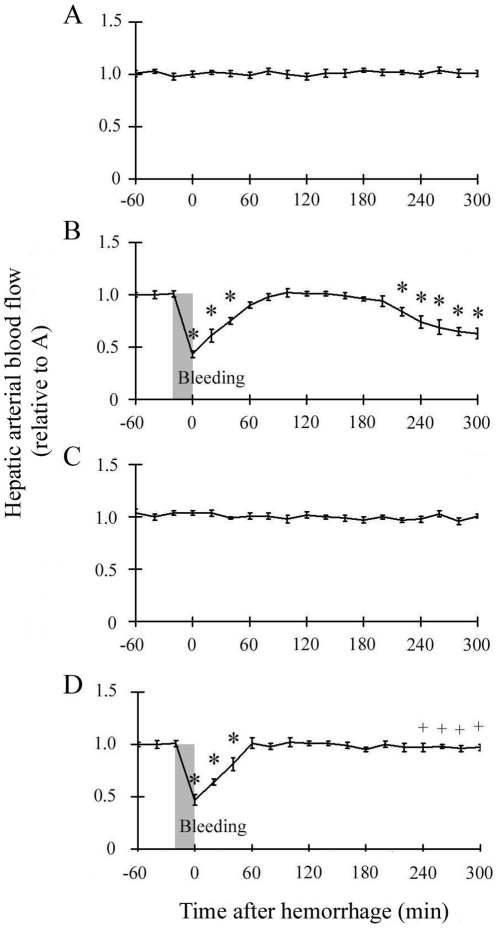
Time course of hepatic arterial blood flow (HBF). The abbreviations are the same as those in Fig. 1. The change in HBF was similar to that in MBP. Data are shown as mean±SE. n = 5/groups, * p<0.05 vs Sham, + p<0.05 between Hemorrhage and FR+Hemorrhage.

Similarly, HBF in the hemorrhage groups decreased after hemorrhage as shown in Fig. 2B and Fig. 2D, (from 1.01±0.03 to 0.43±0.03 in the Hemorrhage group; from 1.01±0.03 to 0.47±0.05 in the FR+Hemorrhage group), and like MBP, HBF recovered thereafter (Fig. 2B, 2D). In the latter phase after hemorrhage, HBF decreased gradually again in the Hemorrhage group (Fig. 2B), but did not show such a decrease in the FR+Hemorrhage group (Fig. 2D).

### Bacterial LPS concentration in the portal vein

**Figure 3:**
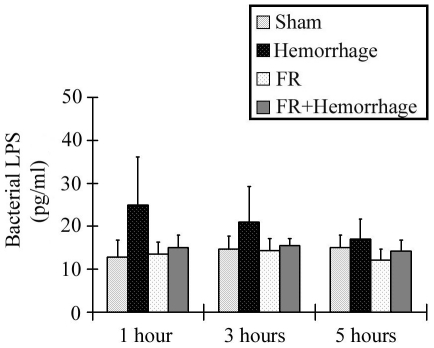
Bacterial LPS concentration in the portal vein. There were no significant increases in the portal LPS concentration in any of the groups throughout the experimental period. Data are shown as mean±SE. n = 5/groups.

There were no significant increases in the portal vein LPS concentration in any of the groups throughout the experimental period as shown in Fig. 3. The portal vein LPS concentration in the Hemorrhage group appeared to increase slightly at 1hour after hemorrhage (25.0±11.1 pg/ml) but did not differ significantly relative to the Sham group (12.8±3.9 pg/ml) throughout the experiment.

### Assessment of p38 MAPK activation in the liver

**Figure 4:**
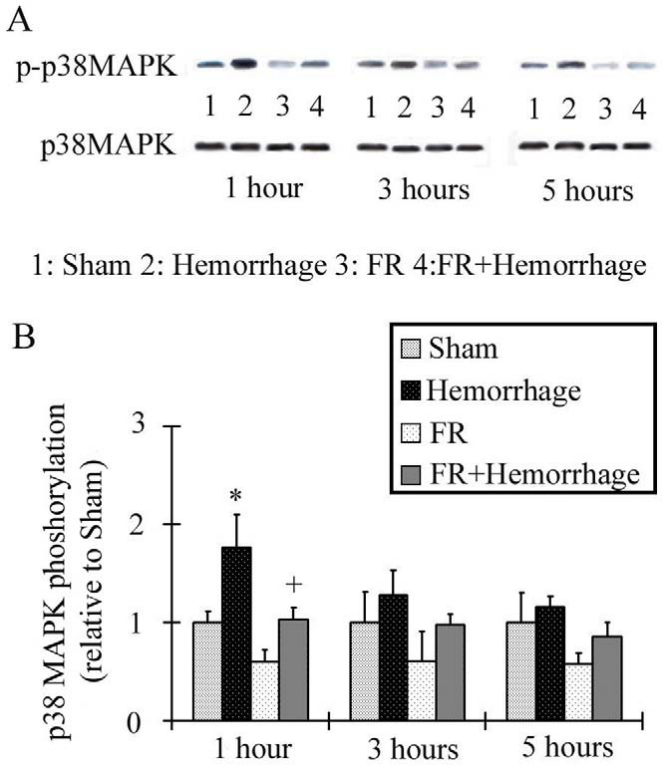
Changes in hepatic p38 MAPK activation after hemorrhage. The activation of p38 MAPK was assessed by Western blotting analysis. p38 MAPK phosphorylation levels, shown on the vertical axis in B, were determined after normalization using the density ratio of the phosphorylated p38 MAPK band (p-p38 MAPK in A) divided by the p38 MAPK band (p38 MAPK in A). Activation of p38 MAPK was significantly higher at 1 hour and returned to the baseline level at 3 hours after hemorrhage. Activation of p38 MAPK activation in the FR+Hemorrhage group did not change significantly at any time after a hemorrhage. Data are shown as mean±SE. n = 5/groups, * p<0.05 vs Sham, + p<0.05 between Hemorrhage and FR+Hemorrhage.

The p38 MAPK phosphorylation level was 1.76±0.34 at 1hour after hemorrhage in the Hemorrhage group, which was significantly higher than in the Sham group, as shown in Fig. 4. Phosphorylation returned to baseline levels at 3 hours after hemorrhage. The phosphorylation of p38 MAPK induced by hemorrhagic shock was inhibited in the FR+Hemorrhage group at 1hour after hemorrhage (1.03±0.12). There were no significant differences from baseline in the p38 MAPK phosphorylation between the Sham group and FR group throughout the experiment.

### TNF-α mRNA expression in the liver and serum TNF-α concentration

**Figure 5:**
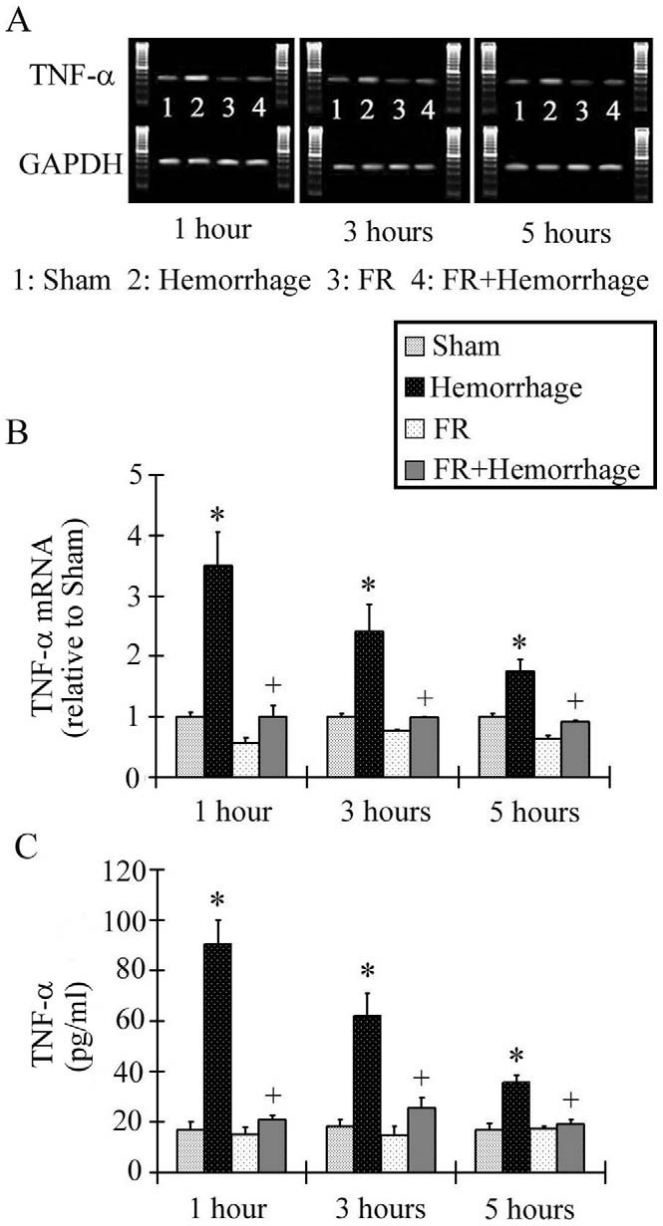
Changes in hepatic TNF-α expression after hemorrhage. TNF-α mRNA was assessed by RT-PCR. Relative TNF-α mRNA expression levels, shown on the vertical axis in B, were expressed after normalization with GAPDH. The Hemorrhage group showed the greatest increase in TNF-α expression at 1 hour post-hemorrhage, and the levels were higher than in the Sham group for up to 5 hours. On the contrary, the TNF-α mRNA level in the FR+Hemorrhage group did not change significantly from the baseline value. The serum TNF-α level in the Hemorrhage group was significantly higher than baseline value at 1 hour after a hemorrhage and decreased subsequently, but was significantly higher than the Sham group at all timepoints post-hemorrhage, as shown in C. In contrast, the serum TNF-α level in the FR+Hemorrhage group did not show an increase from baseline throughout the experiment. Data are shown as mean±SE. n = 5/groups, * p<0.05 vs Sham, + p<0.05 between Hemorrhage and FR+Hemorrhage.

TNF-α mRNA expression in the Hemorrhage group peaked at 1 hour after hemorrhage and was significantly higher than in the Sham group at all times after hemorrhage (3.50±0.55 at 1 hour, 2.40±0.46 at 3 hours, 1.75±0.2 at 5 hours). No increase in TNF-α mRNA expression levels occurred in the FR+Hemorrhage group at any timepoint after hemorrhage (Fig. 5A, Fig. 5B).

The concentration of serum TNF-α is shown in Fig. 5C. The serum TNF-α concentration in the Hemorrhage group was highest at 1 hour after hemorrhage and was significantly higher than in the Sham group at all timepoints (90.5±9.3 pg/ml at 1 hour, 62.0±9.1 pg/ml at 3 hours, 35.5±3.0 pg/ml at 5 hours). The post-hemorrhage increase in serum TNF-α levels was inhibited in the FR+Hemorrhage group at all timepoints (Fig. 5C).

### IL-1β mRNA expression in the liver and serum IL-1β concentration

**Figure 6:**
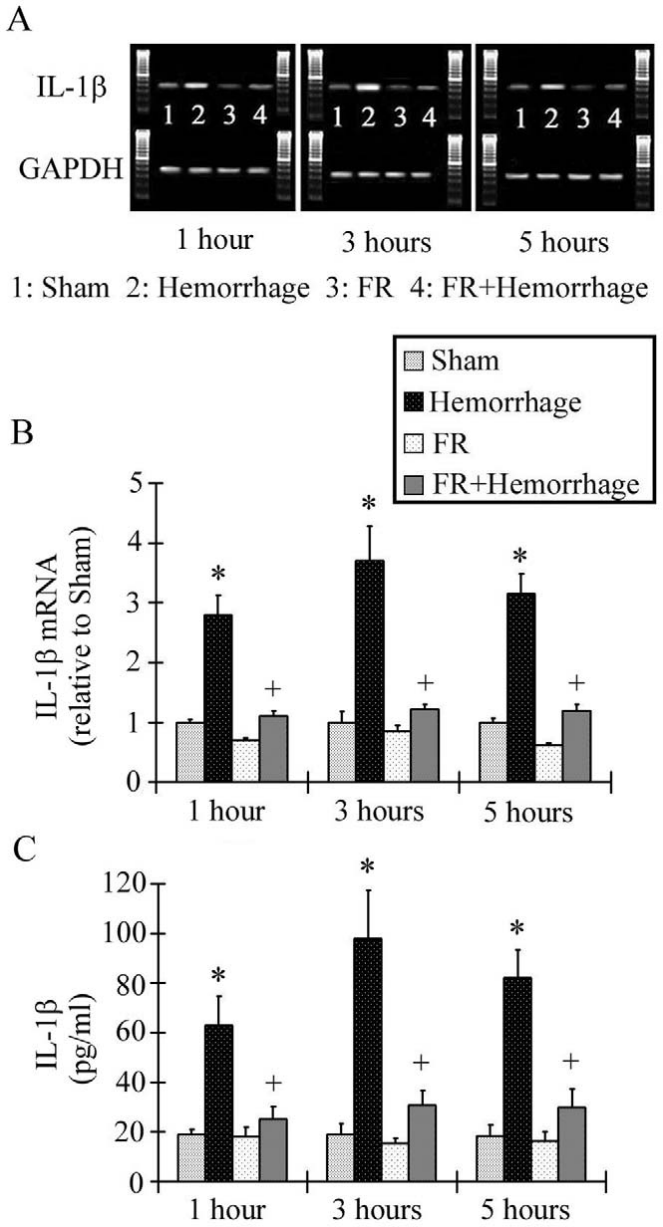
Changes in hepatic IL-1β mRNA expression after hemorrhage. The IL-1β mRNA level in the Hemorrhage group was highest at 3 hours post-hemorrhage, and was higher than that of the Sham group at all timepoints. On the contrary, the IL-1β mRNA level in the FR+Hemorrhage group showed no change from baseline at any timepoint after hemorrhage. Similarly, the serum IL-1β level in the Hemorrhage group was significantly increased from baseline at 3 hours after hemorrhage and then decreased over time, but was significantly higher than in the Sham group at all the timepoints post-hemorrhage as shown in C. In contrast, the serum IL-1β level in the FR+Hemorrhage group showed no change from the baseline value throughout the experiment. Data are shown as mean±SE. n = 5/groups, * p<0.05 vs Sham, + p<0.05 between Hemorrhage and FR+Hemorrhage.

IL-1β mRNA expression in the liver was highest at 3 hours after hemorrhage and was significantly higher than in the Sham group at all timepoints (2.80±0.34 at 1 hour, 3.70±0.58 at 3 hours, 3.2±0.33 at 5 hours). The post-hemorrhage increase in IL-1βmRNA expression levels was suppressed in the FR+Hemorrhage group at all timepoints (Fig. 6A, Fig. 6B).

The serum IL-1βconcentration was also highest at 3 hours after hemorrhage and was significantly higher than in the Sham group at all timepoints (62.9±11.9pg/ml at 1 hour, 97.6±19.7 at 3 hours, 82.4±10.7 at 5 hours). The post-hemorrhage increase in serum IL-1β levels was suppressed in the FR+Hemorrhage group at all timepoints (Fig. 6C).

### Liver enzymes analysis

**Figure 7:**
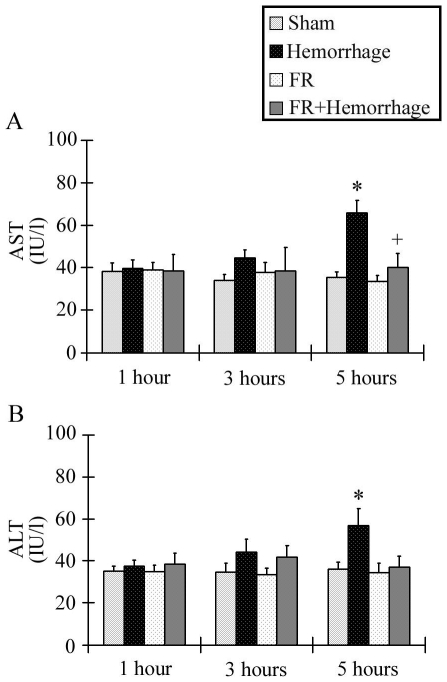
Changes in serum AST and ALT levels. The levels of liver enzymes in the Hemorrhage group increased with time and were significantly higher than in the Sham group at 5 hours after hemorrhage. Administration of FR167653 (FR+Hemorrhage group) inhibited the increase AST and ALT (A and B respectively). Data are shown as mean±SE. n = 5/groups, * p<0.05 vs Sham, + p<0.05 between Hemorrhage and FR+Hemorrhage.

The serum AST level in the Hemorrhage group increased with time and was significantly higher than in the Sham group at 5 hours after hemorrhage (39.6±4.2 IU/l at 1 hour, 44.6±3.8 IU/l at 3 hours, 65.6±6.2 IU/l at 5 hours). In contrast with the Hemorrhage group, the FR+ Hemorrhage group did not show any significant changes in serum AST levels at any timepoint (Fig. 7A). Changes in the serum levels of ALT were similar to those of AST (Fig. 7B). The serum ALT level in the Hemorrhage group increased over time and was significantly higher than the Sham group at 5 hours after hemorrhage (37.4±3.1 IU/l at 1 hour, 44.0±6.5 IU/l at 3 hours, 56.8±8.2 IU/l at 5 hours). There were no significant changes in the serum levels of AST or ALT in the Sham group or FR groups throughout the experimental period.

### Histological examination

**Figure 8:**
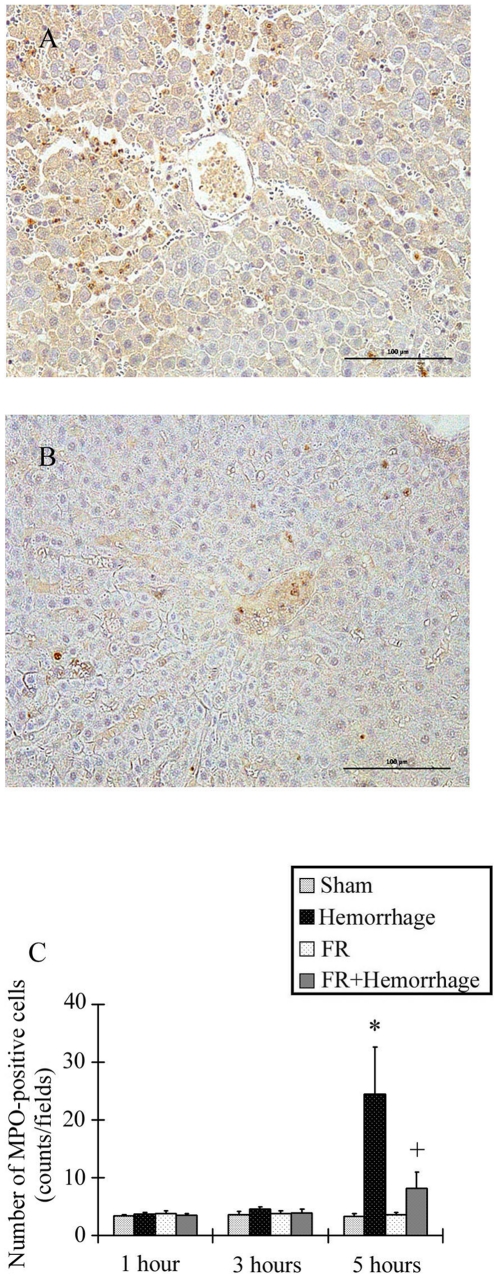
Light micrograph showing the liver at 5 hours post-hemorrhage. The interstitial space was edematous and the sinusoidal capillaries were dilated with diffuse congestion. Some neutrophils, which have the appearance of reddish brown precipitates, were present in the sinusoidal cavity and interstitial space in the Hemorrhage group (A). These histological changes were not observed in the FR+Hemorrhage group (B). Bar = 100 µm. Number of activated neutrophils in the liver (500 µm^2^) as shown in C. In the Hemorrhage group, activated neutrophils were significantly increased at 5 hours, but markedly reduced in the group treated with FR167653 (FR+Hemorrhage) at 5 hours post-hemorrhage. Data are shown as mean±SE. n = 5/groups, * p<0.05 vs Sham, + p<0.05 between Hemorrhage and FR+Hemorrhage.

In the Hemorrhage group, the interstitial space was edematous and the sinusoidal capillaries were dilated with diffuse congestion. A clustered appearance of reddish brown precipitates in the sinusoidal cavity or interstitial space indicated the presence of activated neutrophils (Fig. 8A). These changes were seldom observed in the FR+Hemorrhage group (Fig. 8B). The number of activated neutrophils in the Hemorrhage group (24.5±8.2 counts/field) was significantly higher than in the Sham group (3.4±0.5 counts/field) at 5 hours after hemorrhage (Fig. 8C). In contrast, the number of activated neutrophils in the FR+ Hemorrhage group (8.2±2.8 counts/field) was not significantly higher than in the Sham group and was significantly lower than that of the Hemorrhage group at 5 hours (Fig. 8C).

## Discussion

Several investigators have studied the role of p38 MAPK activation on liver damage following ischemia/reperfusion, dermal burns, and hemorrhage with resuscitation [4], [5], [21], [22]. However, these studies revealed the evidences focusing only on the relationship between liver damage and p38 MAPK activation using pharmacological inhibitors. In this study, we tried to take a more comprehensive approach to analyzing the precise role of p38 MAPK activation in liver damage by identifying related factors associated with the pathological sequence of events, such as blood flow in the hepatic artery, p38 MAPK activation, the activation of pro-inflammatory cytokines, and neutrophil accumulation. The p38 MAPKs are reported to be activated by severe cellular stress such as that due to reactive oxygen species generated from ischemia and reperfusion [4], [23], which are involved in proliferation, differentiation and production of proinflammatory cytokines including TNF-α and IL-1β [7].

Ischemia and/or ischemia-reperfusion is a consequence of the hemodynamic changes that follow hemorrhagic shock [10], [11], [12], [13]. The liver is supplied with approximately 30% of the total cardiac output, 20% of which goes through the hepatic artery and 80% through the portal vein [24], [25]. Both avenues of blood flow depend significantly on the systemic arterial blood pressure [24]. However, once hepatic blood flow decreases following hemorrhagic shock, hypovolemia triggers a corrective response that includes lowering of the resistance in the hepatic artery and the release of endogenous vasodilating substances such as prostaglandins, adenosine, and glucagon [24]. The average oxygen saturation in the portal vein was 74.5% [26], which was significantly lower than that in the hepatic artery [26]. Therefore, we measured blood flow in the hepatic artery in the present study to determine the degree of hepatic ischemia. Hepatic arterial blood flow decreased to approximately 40% of the pre-bleeding level immediately after hemorrhage and temporarily recovered in proportion to the change in systemic arterial pressure (Fig. 1, 2). These results revealed that hemorrhaging of up to 25% of the total blood volume caused an immediate significant ischemic state in the liver. We presumed that the present hepatic ischemia resulting from the decrease in systemic arterial pressure was a primary cause of p38 MAPK activation during the early post-hemorrhage phase (Fig. 4).

Bacterial LPS derived from the gut has also been reported to play an important role in the progression of liver damage through the activation of the p38 MAPK pathway [27]. Jiang et al. suggested that decreased blood flow after hemorrhaging induces intestinal barrier damage followed by the development of bacterial translocation, and demonstrated that plasma bacterial LPS in the portal vein increased at 90 minutes, peaked at 150 minutes and decreased subsequently [28]. In the present study, we measured the plasma concentration of bacterial LPS in the portal vein and although a slight increase was observed in the hemorrhage group, the elevation was not statistically significant (Fig. 3). p38 MAPK was not activated significantly at 3 hours post- hemorrhage (Fig. 4), the time at which the bacterial LPS level had been reported by others to be highest in the portal vein. We concluded that bacterial LPS had little effect on the activation of p38 MAPK in the liver following hemorrhagic shock in our model.

TNF-α and IL-1β in the liver are synthesized mainly by Kupffer cells through the activation of p38MAPK [10], [21], [29], [30]. The expression of TNF-α mRNA and the serum levels of TNF-α peaked at 1 hour post-hemorrhage (Fig. 5), and the expression of IL-1β mRNA and the serum levels of IL-1β peaked at 3 hours (Fig. 6). The temporal pattern of these responses was similar to that in our previous study on renal and cardiac expression of the cytokines after hemorrhagic shock [12], [13]. The peak of p38 MAPK activation was not very robust in comparison with the peak for other pro-inflammatory cytokines. Kobayashi et al. reported that p38 MAPK was significantly activated up to 30 minutes after the induction of reperfusion [4]. Activation of p38 MAPK might have occurred to a greater extent at timepoints earlier than 1 hour after hemorrhage. TNF-α and IL-1β are important factors in the induction of leukocyte chemotaxis in conjunction with adhesion molecules such as β_4_ integrin, ICAM-1, β_2_ integrin, and VCAM-1, which are involved in nutrophil adhesion to the surface of hepatic vascular endothelial cells and neutrophil infiltration in the liver [5], [31], [32]. In this study, neutrophils increased in the liver in the hemorrhage group at 5 hours after hemorrhage (Fig. 8). Adherent neutrophils are known to generate and release numerous active substances, such as proteolytic enzymes and reactive oxygen species [8], [9], [10], and also known to contribute to hepatocyte damage by forming sinusoidal plugs [9], [32]. Activated Kupffer cells also cause hepatocyte damage by releasing reactive oxygen and nitrogen species [10]. All of the above-mentioned factors have the potential to damage both the endothelial layer and adjacent tissue directly or indirectly in the liver. In the present experiments, hepatocyte damage was evaluated based on the serum concentrations of AST and ALT in the late phase after hemorrhagic shock (Fig. 7). FR167653 suppressed inflammation-related liver damage by suppressing p38 MAPK activation. These results demonstrate that activation of p38 MAPK by hepatic ischemia played a key role in the liver dysfunction that occurred as a result of the inflammatory response induced by hemorrhagic shock.

In the case of long term hepatic ischemia followed by reperfusion, p38 MAPK activation enhanced the liver damage via induction of the above-mentioned inflammatory process. On the contrary, p38 MAPK activated as a result of a brief period of ischemia followed by reperfusion was reported to prevent the progression of liver damage induced by a subsequent extended period of ischemia followed by reperfusion, a paradigm referred to as ischemic preconditioning [33], [34]. The present model involved a single 20-minute-bleeding followed by reperfusion in the liver. We could not confirm the efficacy of the preconditioning in this study, and thus the possibility remains that ischemic preconditioning could protect against liver damage following repetitive hemorrhagic shock.

Takahashi et al. reported that FR167653 inhibited p38α MAPK activation selectively [18]. p38α MAPK is highly expressed in leukocytes and endothelial cells, which are readily affected by the decrease of blood flow following hemorrhagic shock. We reasoned that p38α MAPK activation might play a pivotal role in the present liver damage model, though we did not confirm the isoform of p38 MAPK in this study.

In conclusion, the present data indicates that activation of p38 MAPK is essential for the progression of inflammatory hepatic damage following hemorrhagic shock. Thus, inhibition of p38 MAPK activation at an early phase after hemorrhage could be an effective means of preventing the development of liver damage and multiple organ failure following hemorrhagic shock.
